# Unraveling the Neurological Complexity of Polyneuropathy, Organomegaly, Endocrinopathy, Monoclonal Protein, and Skin Changes Syndrome: A Report of a Challenging Case of a Young Woman and Cutting-Edge Advancements in the Field

**DOI:** 10.3390/diseases11040167

**Published:** 2023-11-10

**Authors:** Gioconda Furciniti, Giuseppe Casalino, Francesco M. Lo Russo, Niccolò Bolli, Megi Meneri, Giacomo P. Comi, Stefania P. Corti, Daniele Velardo

**Affiliations:** 1 Department of Pathophysiology and Transplantation, “Dino Ferrari” Center, Università degli Studi di Milano, 20122 Milano, Italy; gioconda.furciniti@unimi.it (G.F.); megi.meneri@unimi.it (M.M.); giacomo.comi@unimi.it (G.P.C.); 2 Eye Clinic, Foundation IRCCS Ca’ Granda Ospedale Maggiore Policlinico, 20122 Milano, Italy; giuseppe.casalino@policlinico.mi.it; 3 Neuroradiology Unit, Foundation IRCCS Ca’ Granda Ospedale Maggiore Policlinico, 20122 Milano, Italy; francesco.lorusso@policlinico.mi.it; 4 Hematology Division, Foundation IRCCS Ca’ Granda Ospedale Maggiore Policlinico, 20122 Milano, Italy; niccolo.bolli@unimi.it; 5 Department of Oncology and Onco-Hematology, Università degli Studi di Milano, 20122 Milano, Italy; 6 Neurology Unit, Foundation IRCCS Ca’ Granda Ospedale Maggiore Policlinico, 20122 Milano, Italy; 7 Neuromuscular and Rare Diseases Unit, Department of Neuroscience, Foundation IRCCS Ca’ Granda Ospedale Maggiore Policlinico, 20122 Milano, Italy

**Keywords:** POEMS syndrome, polyneuropathy, endocrinopathy, M-protein, VEGF

## Abstract

POEMS syndrome—characterized by polyneuropathy, organomegaly, endocrinopathy, M-protein, and skin changes—is an uncommon and complex paraneoplastic disorder encompassing a diverse array of symptoms. Here we report the challenging case of a 34-year-old female who sought medical attention at the emergency department due to distal lower limb weakness. She was breastfeeding her first child at that time. Her condition rapidly deteriorated, making it difficult for her to perform simple tasks independently. Initially, she struggled with activities like jumping or climbing stairs. Eventually, her ability to walk was also compromised. These symptoms underscored the swift evolution of her polyneuropathy. Nerve conduction studies and electromyography confirmed a diagnosis of mixed demyelinating and axonal polyneuropathy. Subsequent investigations, including bone marrow biopsy and immunochemistry testing, revealed a plasma cell disorder characterized by lambda monoclonal gammopathy, along with elevated levels of vascular endothelial growth factor (VEGF > 8000 pg/mL). This pivotal finding led to the diagnosis of POEMS syndrome, prompting the initiation of antineoplastic therapy (daratumumab-lenalidomide-dexamethasone) to manage this condition. An autologous cell transplantation was planned. The rarity of POEMS syndrome and its diverse clinical manifestations often lead to an incorrect or delayed diagnosis. Our case underscores the importance of considering this syndrome in patients presenting with acute or subacute polyneuropathy, even if the patients are young. In conclusion, this case elucidates the diagnostic complexities of POEMS syndrome, emphasizing the integral role of comprehensive multidisciplinary evaluations and the potential influence of increased VEGF as a diagnostic key element and possible therapeutic target.

## 1. Introduction

POEMS syndrome, a rare and enigmatic paraneoplastic disease, has a prevalence of approximately 0.3 cases per 100,000 [1]. Its name is an acronym for the key features of this syndrome: polyneuropathy, organomegaly, endocrinopathy, monoclonal protein, and skin changes. To diagnose POEMS syndrome, the presence of two essential factors is required—polyneuropathy and monoclonal plasma cell disorder. Its diagnosis requires the presence of at least one major criterion (sclerotic bone lesion, increased vascular endothelial growth factor (VEGF) levels, and/or Castleman disease) and one minor criterion (organomegaly, endocrinopathy, characteristic skin changes, papilledema, extravascular volume overload, and/or thrombocytosis/polycythemia) [1].

The pathophysiology of POEMS syndrome is only partly understood. Elevated VEGF levels have emerged as a crucial element, such that VEGF can be used as a diagnostic marker, a means to monitor disease activity, and potentially a therapeutic target [2]. VEGF may play a pivotal role in disrupting the blood–nerve barrier, leading to demyelination and axonal damage, and thereby contributing to the observed neuropathy [3].

POEMS syndrome presents with a wide array of symptoms that fit the acronym “POEMS”, accompanied by other nonspecific signs such as ascites, pleural effusion, and thrombocytosis. This vague and diverse presentation commonly leads to under-recognition or delayed diagnosis of this condition. In some cases, patients might be misdiagnosed with a primary neurologic disorder, such as chronic inflammatory demyelinating polyradiculoneuropathy or, rarely, subacute demyelinating polyradiculopathy [1].

For general practitioners, it is crucial to recognize the signs and symptoms of POEMS syndrome, such as progressive weakness, sensory abnormalities, and a collection of systemic symptoms like organomegaly or skin changes, despite its rarity. Early suspicion and referral to a specialized center for confirmatory testing, including blood tests for monoclonal protein and VEGF levels, and imaging for sclerotic bone lesions, are essential for the timely diagnosis and initiation of appropriate therapy.

Here we report a case with a unique presentation of POEMS syndrome. A young woman experienced unusually rapid and progressive neuropathy, accompanied by a distinct array of multisystemic features, eventually leading to the diagnosis of POEMS syndrome. In this paper, we thoroughly explore the diagnostic approach and therapeutic strategies used to manage this intricate condition. Given the knowledge gaps regarding POEMS syndrome, there is a steadfast call for additional research, as there is a pressing need to deepen our comprehension of this complex and serious condition. Continuous investigation is imperative to improve diagnosis and treatment methods, which will ultimately lead to better outcomes for patients.

## 2. Case Report

A 34-year-old woman was admitted to our hospital with a one-month history of gradually developing weakness in the distal lower limbs, along with dysesthesias and paresthesias. Symptoms notably began about two months postpartum. At the time of her first neurological assessment, she was experiencing significant difficulty climbing stairs and driving a car and required assistance when walking. She also reported blurred vision, accompanied by throbbing morning headaches. Physical examination revealed hyperpigmentation on her face and neckline and acrocyanosis of the upper limbs.

Prior to the emergency of the neurological symptoms, the patient had experienced episodes of diarrhea, abdominal pain, weight loss, fatigue, and intermittent night fevers. Infectious diseases were excluded during previous evaluations.

The initial neurological examination revealed mild distal-predominant weakness in the lower limbs, reduced vibratory and position sense, and a loss of tendon reflexes. Nerve conduction studies and electromyography demonstrated severe mixed axonal and demyelinating polyneuropathy, affecting both motor and sensory nerves in all four extremities, with mild signs of active denervation in the distal muscles of the lower limbs (Table 1). 

During hospitalization, the patient’s lower limb muscle weakness progressively worsened, eventually rendering her unable to move her feet, with a predominance of weakness in the right lower limb. Further blood tests revealed thrombocytosis, as well as the presence of serum and urine immunoglobulin A (IgA)-lambda monoclonal gammopathy. The patient also exhibited elevated serum and urine free-light chains, plasma beta-2 microglobulin (Table 2), and subclinical hypothyroidism.

**Figure 1:**
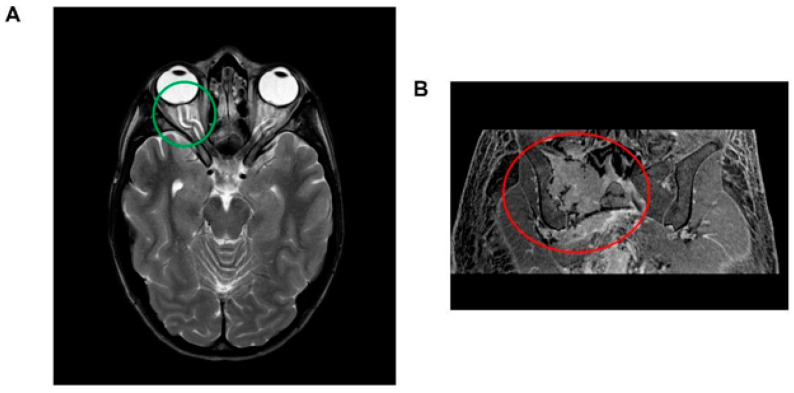
(**A**) Brain magnetic resonance imaging (MRI) illustrating indirect indicators of intracranial hypertension. Notable features include the tortuosity of the optic nerve accompanied by sheath enlargement and the intraocular protrusion of the optic nerve head, which is coupled with posterior scleral flattening (highlighted within the green circle). Additionally, a significant enhancement of the optic disc is observed. (**B**) Abdominal magnetic resonance imaging (MRI) depiction of a lesion located at the right hip, exhibiting transverse diameters of 70 × 66 mm. The lesion is characterized by a composition of both osteolytic and osteosclerotic components (encircled in red).

Brain magnetic resonance imaging (MRI) revealed indirect signs of intracranial hypertension, including optic nerve tortuosity with sheath enlargement and intraocular protrusion of the optic nerve head, along with flattening of the posterior sclera. Significant enhancement of the optic disc was also observed (Figure 1A). Angio-MRI findings revealed distinct constriction in the dural venous sinuses around the transverse and sigmoid sinus junctions, which is a trait observed in approximately 90% of individuals with suspected idiopathic intracranial hypertension. Other potential causes—such as secondary intracranial hypertension, venous sinus thrombosis, and parenchymal lesions—were carefully excluded. Fundoscopic examination confirmed bilateral papilledema.

Abdominal MRI revealed hepatosplenomegaly, pelvic free fluid, and a sizable lesion (transverse diameters 70 × 66 mm) in the right hip bone (Figure 1B). A whole-body computed tomography (CT) scan showed that the hip bone lesion had mixed characteristics—including both osteolytic and osteosclerotic components—and was associated with other lesions in both the ipsilateral and contralateral hip bones. A CT-guided biopsy of the lesion confirmed plasma cell dyscrasia. Staging investigations—including superficial and deep lymph node ultrasounds and bone marrow histological and cytological examinations—revealed normal findings. A positron emission tomography scan confirmed high glucose uptake in the hip bone lesion. Notably, the patient exhibited significantly elevated serum VEGF levels (>8000 pg/mL). Based on these clinical and laboratory findings, a diagnosis of POEMS syndrome was established.

Initially, the patient was considered for immunomodulatory therapy, followed by high-dose chemotherapy with autologous peripheral blood stem cell transplantation. However, she was deemed temporarily ineligible for transplantation due to her reduced performance status. Therefore, antineoplastic therapy with daratumumab-lenalidomide-dexamethasone was initiated. At present, the patient’s clinical condition remains stable, and a cell transplantation is planned. Further follow-up is required to fully assess the outcome of the administered therapy.

## 3. Discussion

POEMS syndrome is a rare multisystem disorder characterized by a constellation of symptoms and has the potential to cause significant morbidity and mortality if left untreated. A study conducted at the Mayo Clinic included 99 patients with POEMS syndrome and revealed that the median age of onset was 51 years (range, 30–83 years), with approximately 63% of affected individuals being male [4]. Although POEMS syndrome is more commonly reported in older individuals, a few case reports have involved younger patients, with two of the younger patients being male and four being female, including our present case [5,6,7,8,9]. Notably, four of these younger patients have exhibited an excellent response to systemic therapy, showing improvements in both ocular and systemic symptoms within 4–6 months after treatment [5,6,7,8,9]. Therefore, systemic therapy commonly yields promising results in young patients with POEMS syndrome.

Ocular manifestations are observed in a variable percentage of POEMS syndrome cases, with papilledema being the most common, present in 52% of patients [10]. Papilledema is generally considered a late finding in the disease, with only four cases reported in the literature where papilledema was among the initial symptoms [5,7,8,9]. Therefore, our present case can be considered atypical because the patient experienced blurred vision during the initial phase of the disease. Interestingly, recent studies have described a link between optic disc edema and serum VEGF levels, with evidence of edema improvement after VEGF is reduced through systemic therapy [11].

Polyneuropathy is a consistent feature of POEMS syndrome and is the initial symptom in over 60% of cases [12]. Neuropathy most often progresses chronically; however, our patient exhibited an unusually rapid progression, losing her ability to walk just two months after symptom onset, which differs from the progression typically reported in the literature [6]. Electrodiagnostic studies commonly demonstrate length-dependent sensory-motor polyneuropathy, often with a demyelinating pattern and minimal axonal damage [6]. Nevertheless, some cases, such as ours, exhibit a mixed pattern with prominent axonal damage. Axonal polyneuropathy is relatively rare in POEMS syndrome, with only two cases reported to date—one showing pure acute axonal polyneuropathy and the other exhibiting a mixed neurophysiological pattern with predominant axonal damage [13,14].

Furthermore, bone lesions occur in a varying percentage of POEMS syndrome patients (45–95%) [15]. The pelvis stands out as the most commonly affected site (71.1%), followed by the spine (47.3%), skull (36.5%), and extremities (28.9%) [15]. Multiple bone lesions are frequently detected, accounting for 68% of cases [6]. Among the different types of bone lesions, osteosclerotic lesions are the most common (97% of cases), distantly followed by mixed osteosclerotic-osteolytic (2%) and osteolytic (0.6–1%) types [16]. Interestingly, bone lesions often manifest without symptoms; patients typically experience bone pain only with lesions of osteolytic or mixed nature [15]. In the context of our present case, it must be emphasized that while osteosclerotic lesions are commonly associated with POEMS syndrome, mixed lesions are also prevalent and can remain asymptomatic for an extended duration, regardless of their size. Intriguingly, no significant correlation has been established between the number or type of bone lesions and patient survival [3].

Given the complex clinical nature of POEMS syndrome, the importance of conducting a thorough and detailed differential diagnosis cannot be overstated. In neurological evaluations, it is critical to explore various potential conditions that could present with neuropathy, especially those associated with monoclonal proteins. In such cases, one frequently considered disease is CIDP, which may coexist with monoclonal gammopathy. A previous study demonstrated that patients with POEMS syndrome tend to complain of pain, which might be a key for distinguishing POEMS from CIDP [17]. Another significant syndrome to consider is chronic atrophic neuropathy with ophthalmoplegia, M-protein, cold agglutinins, and disialosyl antibodies (CANOMAD), which also involves the coexistence of monoclonal proteins. It is essential to differentiate these conditions from other plasma cell dyscrasias, such as amyloidosis, which can also manifest with neuropathy. Before initiating therapy, it is of utmost importance to confirm whether the patient meets the criteria for plasma cell myeloma. Although the therapeutic options may be similar for both POEMS syndrome and myeloma, treatment decisions should be influenced by the consideration that myeloma therapy may carry a higher potential for toxicity in patients with POEMS syndrome [2,18,19].

Therapeutic approaches differ between limited- and advanced-stage diseases [2,18,19]. In POEMS syndrome, the limited-stage disease describes cases where bone marrow biopsies reveal the absence of clonal plasma cells and only 1–2 lesions are present. For patients in this stage, treatment typically includes involved-field radiation therapy at specific doses. Notably, a study conducted at the Mayo Clinic demonstrated that patients with limited-stage disease had a remarkable 4-year overall survival rate of 97% and an event-free survival rate of 52% [2,18,19].

**Figure 2:**
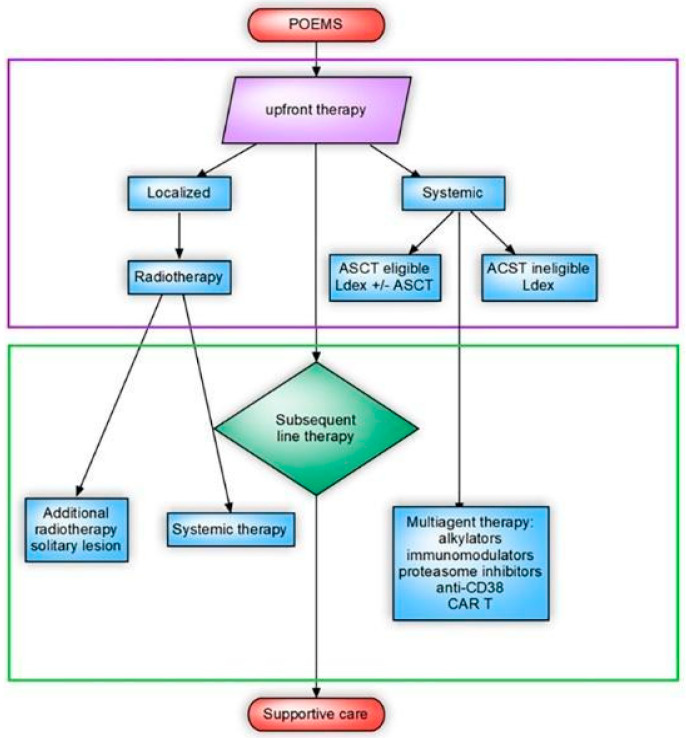
A comprehensive flow chart delineating the recommended therapeutic treatment protocol for polyneuropathy, organomegaly, endocrinopathy, monoclonal gammopathy, and skin changes (POEMS) syndrome. The illustrated treatment algorithm encompasses two pivotal components: autologous stem cell transplantation (ASCT), which is a process that involves reinfusing the patient’s own stem cells to regenerate healthy bone marrow, is a crucial aspect of the treatment strategy; combination treatment with lenalidomide and dexamethasone (Ldex Treatment), which is an aspect of the treatment that involves the concurrent administration of lenalidomide and dexamethasone, works synergistically to manage the symptoms and progression of POEMS syndrome.

For advanced-stage POEMS syndrome, there are no established treatment guidelines, and the therapeutic approaches used for myeloma are often applied (Figure 2). Neurologic responses to treatment may be observed after approximately 6 months, with the maximum effect becoming evident after 2–3 years. It can also take a considerable amount of time for treatment effects to become evident on PET-CT, typically 6–12 months. However, other symptoms may improve earlier. Although the clinical response is associated with an enhanced quality of life, the attainment of hematological remission plays a pivotal role in shaping patient outcomes. Notably, those who achieve hematological complete response (CR) experience the most significant progression-free survival (PFS), with an impressive 88% 6-year PFS rate [2,18,19].

Over the last two decades, the primary treatments for eligible patients with POEMS syndrome have included high-dose melphalan and autologous stem cell transplantation (ASCT). High-dose melphalan is used as a conditioning regimen for ASCT. Performing induction therapy before ASCT can lead to faster hematological and organ responses, positively impacting PFS. Induction therapy also reduces the occurrence of engraftment syndrome and enables the performance of ASCT in asymptomatic patients, such that it has become a preferred approach over ASCT alone. The percentage of ASCT performed with induction treatment has significantly increased over the years [2,18,19].

Despite the availability of novel drugs, low-dose alkylator-based therapy continues to be considered a viable treatment option. Research studies have highlighted the potential of melphalan, an alkylating agent, and dexamethasone (Mdex) for providing partial relief from symptoms, with approximately 38% of treated patients achieving a hematological complete response (CR) [2,18,19].

Immunomodulatory agents, such as lenalidomide, have become common as both first-line and second-line treatments. Lenalidomide is chemically related to thalidomide and has immunomodulatory action. Lenalidomide with dexamethasone (Ldex) has yielded positive effects, with response rates comparable to those achieved with ASCT. The decision between ASCT or a lenalidomide-based regimen depends on the individual patient’s circumstances, and securing stem cells early may be important for high-risk patients [2,18,19].

Proteasome inhibitors, such as bortezomib, have shown effectiveness in treating POEMS syndrome. Bortezomib, by inhibiting the proteasome, exerts stress on cancer cells, leading to their death. Bortezomib combined with dexamethasone (Veldex) yields quick symptomatic improvement, with reversible neuropathy observed in only a small percentage of patients. Bortezomib combined with cyclophosphamide and dexamethasone (VCD) has also been used effectively, and three-drug therapies based on bortezomib have been successful in subsequent lines of treatment. Ixazomib, a proteasome inhibitor with no neurotoxicity, also shows promise in early observations. Moreover, carfilzomib-based regimens may also be effective and safe for resistant/relapsed disease cases [20]. Overall, treatment decisions for POEMS syndrome should be tailored to the individual patient, taking into consideration the disease stage, response to therapy, and potential side effects of treatment.

Recent research has explored several promising new treatment avenues for POEMS syndrome. Among them, anti-CD38 (an antigen expressed by myeloma cells) monoclonal antibodies, such as daratumumab, have emerged as a compelling option. These antibodies have demonstrated notable effectiveness in treating multiple myeloma and are showing promise as a potential therapy for POEMS syndrome. Limited case reports have highlighted the combination of daratumumab with lenalidomide as an upfront therapy for POEMS syndrome, with findings suggesting a potential therapeutic benefit. Furthermore, case reports have demonstrated the efficacy of daratumumab, both as a stand-alone therapy and in combination with other anti-myeloma drugs, for patients with relapsed/refractory POEMS syndrome. There remains a need for more comprehensive and in-depth research efforts to fully understand the potential of anti-CD38 antibodies in addressing this complex syndrome [2,18,19].

Another intriguing area of investigation revolves around CAR T-cell-based therapy for POEMS syndrome. The presently available data are limited to a single case report showing the efficacy of B-cell maturation antigen (BCMA) therapy in a patient with both POEMS syndrome and myeloma. BCMA is a protein that is primarily expressed on the surface of plasma cells, including myeloma cells. CAR T-cells targeting BCMA have shown promise in the treatment of multiple myeloma because they can recognize and kill BCMA-expressing cancer cells. Additional research is needed to explore the potential of CAR T-cell-based therapy as a viable treatment modality for POEMS syndrome [21].

Regarding anti-VEGF therapy, conflicting findings have been reported about the efficacy of bevacizumab for treating POEMS syndrome. Bevacizumab is a monoclonal antibody that specifically binds and inhibits the biological activity of human VEGF. Nonetheless, there are indications that intravitreal administration of bevacizumab may offer benefits in patients with optic edema associated with POEMS syndrome, as documented in individual case reports [2,18,19]. Further research is needed to clarify the potential role of bevacizumab in the overall treatment strategy for this syndrome.

Overall, more extensive research and clinical trials are needed to fully evaluate the effectiveness and safety of new therapeutic avenues for treating this rare and complex condition. The quest to unravel the mysteries surrounding POEMS syndrome demands continued scientific inquiry and dedicated efforts to improve treatment outcomes for those affected by this challenging condition.

Regarding our presented case, the patient’s initial planned treatment involved immunomodulatory therapy followed by high-dose chemotherapy, along with autologous blood stem cell transplantation. However, due to her decreased performance status, she was temporarily considered unfit for the transplantation procedure. Therefore, the hematological medical team decided to start antineoplastic therapy using daratumumab-lenalidomide-dexamethasone. Currently, the patient’s clinical condition is stable, and there are plans to proceed with ASCT once she is deemed eligible.

The present clinical case allows us to expand the phenotypic features of central and peripheral neurological disorders present at the onset of POEMS syndrome in young patients and emphasizes the importance of proper differential diagnosis for treatment purposes. The most striking finding is the progression speed of the neuropathy, which has already been observed in the literature in rare cases [6]. We speculate that this finding could be attributed to the involvement of both myelin and axon from the beginning and the localization of the hip bone lesion close to the lumbar plexus, with possible additional mechanical damaging effects.

A constraint of the case series is that it involves a single patient and has a brief follow-up period, making it impossible to furnish comprehensive information regarding the benefits and potential risks of treatment for this category of patients. Close monitoring and additional follow-up will be required to fully evaluate the outcome of the therapy.

Early diagnosis and prompt treatment are crucial because they can prevent clinical progression and offer patients a better chance of recovery. Further research and case studies are warranted to enhance our understanding of this complex and heterogeneous syndrome. In conclusion, although POEMS syndrome is a rare diagnosis, it should be considered in patients presenting with papilledema and acute or subacute axonal neuropathy, along with multisystemic findings.

## Figures and Tables

**Table 1:** Nerve conduction studies (top tables) and needle electromyography (bottom table) findings during hospital admission, showing severe mixed axonal and demyelinating polyneuropathy with active denervation in distal leg muscles. CMAP, compound muscle action potential; SNAP, sensory nerve action potential.

Motor Conduction Study	CMAP Amplitude (mV)	CMAP Duration (ms)	Distal Latency (ms)	Conduction Velocity (m/s)	F Wave Latency (ms)
Ulnar nerve	7.4	7.4	1.7	35.4	38.7
Tibial nerve	-	-	-	-	-
Common peroneal nerve	0.3	13	4.4	33.3	4.4
Sensory conduction study		SNAP amplitude (μV)		Conduction velocity (m/s)	
Ulnar nerve		5.4		41.7	
Sural nerve		6		35.5	
Muscle		Spontaneous activity		Recruitment	
Tibialis anterior (right)		Fibrillation (2+)		Reduced	
Tibialis anterior (left)		Fibrillation (2+)		Reduced	
Vastus medialis		-		Reduced	
Interosseus		-		Reduced	

**Table 2:** Results of relevant laboratory tests performed at the time of hospital admission.

Laboratory Test	Results	Normal Values
Platelets	180.000/microL	150.000–450.000/microL
Sieric IgA	924 mg/dL	70–400 mg/dL
Sieric kappa/lambda ratio	0.20	0.26–1.65
Bence Jones proteinuria	Present (IgA lambda)	Absent
Beta2-microglobulin	3.9 mg/dL	0.8–2.2 mg/dL
TSH	8.22 μU/mL	0.2–4.3 μU/mL
fT3	1.9 ng/L	2.0–5.0 ng/L

## Data Availability

Data are available upon request.
